# Effects of a Single Bout of Resistance Exercise in Different Volumes on Endothelium Adaptations in Healthy Animals

**DOI:** 10.5935/abc.20170060

**Published:** 2017-05

**Authors:** Marcelo Mendonça Mota, Tharciano Luiz Teixeira Braga da Silva, Fabricio Nunes Macedo, Thássio Ricardo Ribeiro Mesquita, Lucindo José Quintans Júnior, Valter Joviniano de Santana-Filho, Sandra Lauton-Santos, Márcio Roberto Viana Santos

**Affiliations:** 1 Universidade Federal de Sergipe, Aracaju, SE - Brazil; 2 Curso de Educação Física da Faculdade Estácio de Sergipe (Estácio/FASE), Aracaju, SE - Brazil

**Keywords:** Exercise, Endothelium, Physical Conditioning, Animal, Muscle, Smooth, Vascular, Nitric Oxide, Vasodilatation, Rats

## Abstract

**Background:**

Resistance exercise (RE) has been recommended for patients with cardiovascular diseases. Recently, a few studies have demonstrated that the intensity of a single bout of RE has an effect on endothelial adaptations to exercise. However, there is no data about the effects of different volumes of RE on endothelium function.

**Objective:**

The aim of the study was to evaluate the effects of different volumes of RE in a single bout on endothelium-dependent vasodilatation and nitric oxide (NO) synthesis in the mesenteric artery of healthy animals.

**Methods:**

Male Wistar rats were divided into three groups: Control (Ct); low-volume RE (LV, 5 sets x 10 repetitions) and high-volume RE (HV, 15 sets x 10 repetitions). The established intensity was 70% of the maximal repetition test. After the exercise protocol, rings of mesenteric artery were used for assessment of vascular reactivity, and other mesenteric arteries were prepared for detection of measure NO production by DAF-FM fluorescence. Insulin responsiveness on NO synthesis was evaluated by stimulating the vascular rings with insulin (10 nM).

**Results:**

The maximal relaxation response to insulin increased in the HV group only as compared with the Ct group. Moreover, the inhibition of nitric oxide synthesis (L-NAME) completely abolished the insulin-induced vasorelaxation in exercised rats. NO production showed a volume-dependent increase in the endothelial and smooth muscle layer. In endothelial layer, only Ct and LV groups showed a significant increase in NO synthesis when compared to their respective group under basal condition. On the other hand, in smooth muscle layer, NO fluorescence increased in all groups when compared to their respective group under basal condition.

**Conclusions:**

Our results suggest that a single bout of RE promotes vascular endothelium changes in a volume-dependent manner. The 15 sets x 10 repetitions exercise plan induced the greatest levels of NO synthesis.

## Introduction

Physical activity induces physiological adaptations of the endothelium, contributing to the local control of vascular tone.^1^ Besides, the beneficial effects of regular exercise on sympathetic and parasympathetic tone,^2^ blood coagulation,^3^ myocardial contractility^4^ and release of endothelium-derived relaxing factors^5^ are likely to improve cardiovascular health and reduce the risk of diseases.

Exercise training is nowadays one of the main non-pharmacological tool in the maintenance of a healthy life. Its effects involve recurrent exposure to changes in cardiovascular hemodynamics promoted by bouts of physical activity. In response to acute exercise, numerous intra- and extracellular pathways are activated to increase blood flow to active muscles.^6,7^ At the onset of exercise, the mechanical action of skeletal muscle creates a 'muscle-pump', which causes an immediate increase in blood flow.^7,8^ This abrupt change in blood flow towards the exercised tissues, promotes a reduction in blood supply in visceral region, phenomena known as exercise hyperemia.^7,8^

Many evidences suggest that striation in the vascular wall, associated with pulsatile flow, intermittent hypoxia and release of catecholamines are essential factors for nitric oxide (NO) production during a single bout of exercise.^9-11^ Recently, our group have showed that acute resistance exercise induce an intensity-dependent effect on NO synthesis and vascular relaxation in mesenteric arteries of healthy rats.^12,13^ The mesenteric artery regulates 20% of the blood flow and effectively participates in the total peripheral resistance, and thus, is directly involved in vascular changes promoted by exercise.^9^

In addition, our group has recently demonstrated a strong, positive relationship between the magnitude of vascular adaptations to exercise and exercise intensity.^12^ Nevertheless, exercise prescription depends on two different aspects - intensity and volume of exercise. The volume of exercise directly affects the demand of oxygen and other nutrients in attempt to recover from the stress promoted by consecutive muscle contractions. Thus, it is expected that changes in training volume promote different vascular adaptations, i.e., the higher the volume of exercises the higher the metabolic demand.

In addition, there are no studies investigating acute vascular adaptations to different volumes of resistance exercise. This information could guide the prescription of long-term training in cardiovascular disease conditions. Thus, our study aimed to evaluate the influence of the volume of resistance exercise on endothelium-dependent vasodilatation and NO synthesis in mesenteric artery of healthy animals.

## Methods

### Animals

Twenty-four male Wistar rats (250-350 g, 8-10 weeks old) were used for all experiments. The rats were randomized into three groups: control (Ct, n = 8), low-volume (LV, n = 8) and high-volume resistance exercise (HV, n = 8). All procedures were in agreement with the Brazilian Society of Laboratory Animal Sciences and were approved by the Ethics Committee on Animal Research XXX (omitted to the review process), Brazil (protocol # 80/2010).

### Resistance exercise protocol

Animals were exercised following a model described by Tamaki et al.^14^ Electrical stimulation (20 V, 0.3 s duration, at 3 s intervals) was applied on the tail of the rat through a surface electrode. The animals underwent three days of familiarization; firstly, they were placed on the apparatus and left on exercise position for 5 min to reduce the stress caused by the equipment and handling of the animals. After the familiarization period, the animals performed one maximum repetition (1RM) test, which consisted of determining the maximum lifted load in one repetition. After 2 days, the animals underwent the protocol of leg extension exercise - 5 (LV) or 15 (HV) sets with 10 repetitions and a 180s resting period between each set. The animals exercised in intensity of 70% of 1RM. Animals of the Ct group were maintained under the same conditions of the LV and HV animals but at resting position.

### Vascular reactivity studies

Immediately after exercise, the animals were sacrificed. The superior mesenteric artery was removed, stripped of connective and adipose tissues, and sectioned into rings (1-2 mm). Rings were suspended in organ baths containing 10 mL of Tyrode's solution by fine stainless steel hooks connected to a force transducer (Letica, Model TRI210; Barcelona, Spain) with cotton threads. This solution was continually gassed with carbogen (95% O_2_ and 5% CO_2_) and maintained at 37°C and the rings maintained at a resting tension of 0.75 g for 60 min (stabilization period). The functionality of the endothelium was assessed by the ability of acetylcholine (ACh, 1 *µ*M; Sigma-Aldrich, USA) to induce more than 75% relaxation of precontracted vascular rings with phenylephrine (Phe, 1 *µ*M; Sigma-Aldrich, USA). After that, changes in vascular reactivity were assessed by obtaining concentration-response curves for insulin (Novo Nordisk, Bagsvaerd, Denmark) (10^−13^-10^−6^ M). The rings were then washed out and new insulin-induced relaxation was obtained after incubation with a non-specific inhibitor of nitric oxide synthase, L-N^G^-Nitroarginine methyl ester (L-NAME, 100 *µ*Mol/L; Sigma-Aldrich, USA), for 30 min. This was used to evaluate the role of NO on insulin-induced vascular relaxation.

### Measurement of NO Production

NO production in mesenteric artery ring was determined by using a fluorescent cell permeable dye for NO, DAF-FM (4,-amino-5 methylamino-2',7'-diaminofluorescein diacetate, Molecular Probes, USA), as previously described.^15^ In order to detect NO, freshly isolated mesenteric artery was loaded with 10 *µ*M of the probe for 40 min at 37°C. Twenty minutes after the onset of the probing, some rings were stimulated with 10 nM of regular human insulin for 20 min and then washed for 40 min with Tyrode´s solution. Mesenteric segments were frozen and cut into 20*µ*m-thick sections. Images were recorded using a fluorescence microscope (IX2-ICB, Olympus^®^, USA) under identical settings. The fluorescence intensity was measured using ImageJ software (NIH, USA). A minimum of ten regions were randomly selected from the endothelial and smooth muscle layers of each mesenteric section. It is worth to note that smooth muscle exhibits an autofluorescence, therefore, in order to avoid misleading fluorescence measurements, analyses of images were carefully performed selecting the region of interest within the smooth muscle fibers.

### Statistical Analysis

Initially, all data underwent the Kolmogorov-Smirnov test to determine whether the probability distributions were parametric or non-parametric. All data had normal distribution. The values were expressed as mean ± standard error of the mean (SEM). One-way analysis of variance (ANOVA) followed by the Bonferroni`s test were performed using GraphPad Prism Software (San Diego, CA, USA). The NO fluorescence microscopy images were analyzed according to the intensity of the fluorescence per normalized area, represented in arbitrary unit (a.u.). The values were considered statistically significant when p < 0.05.

## Results

### Acute effect of different resistance exercise volumes on endothelium-dependent vasodilation

**Figure 1:**
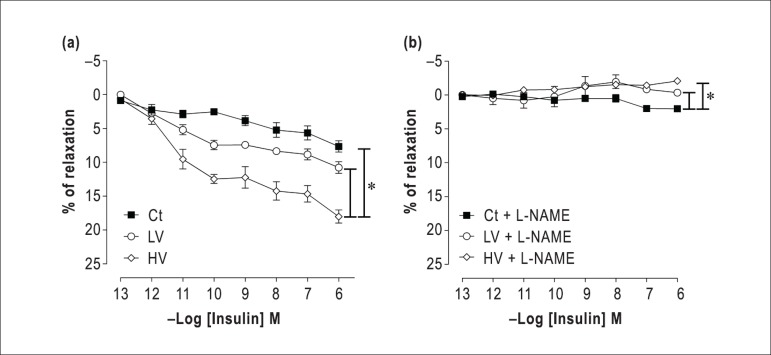
Effects of a single bout of resistance exercise in different levels of volume on endothelium-dependent relaxation. (a) Concentration-response curves for insulin (10^-13^ - 10^-6^M) in rings isolated from the superior mesenteric artery with functional endothelium and pre-contracted with phenylephrine (Phe) (1 *µ*M). (b) Concentration-response curves for insulin in rings pre-incubated with nitric oxide inhibitor (L-NAME: 100 *µ*M). Ct: control, LV: low-volume and HV: high-volume. Statistical differences were determined by one-way ANOVA followed by Bonferroni's test. Results are expressed as mean ± SEM. *p < 0.05.

As shown in the Figure 1a, in all groups, insulin caused a concentration-dependent vasodilation in superior mesenteric arteries. Despite the tendency to increase the insulin-induced vasodilation in LV group, no significant difference was found in comparison to the Ct group (Figure 1a). However, resistance exercise at HV significantly increased the relaxation induced by insulin compared to Ct and LV groups (Figure 1a). The effect of NOS on insulin-induced vascular relaxation was evaluated in the presence of L-NAME. As shown in the Table 1, insulin-induced vasodilation was significantly reduced in Ct group; however, in both exercised groups the vasodilation was completely abolished showing a significant reversion of the curve when compared to Ct. No statistically significant differences were observed between the LV and HV groups. (Figure 1b).

**Table 1:** Values of Rmax obtained from concentration-response curves for insulin in mesenteric arteries before and after incubation with L-NAME

Groups	Insulin (%)	Insulin + L-NAME (%)
Control	7.66 ± 0.83	2.01 ± 0.24^§^
Low volume	10.77 ± 0.86	–0.36 ± 0.36^§,†^
High volume	18.01 ± 0.97^*, #^	–2.08 ± 0.19^§,†^

The experiments were performed in the absence of L-NAME (insulin) and in the presence of 100 µM of L-NAME (L-NAME). Statistical differences were determined by one-way ANOVA followed by the Bonferroni post-hoc test. The data are expressed as mean ± SEM.

* p < 0.05 vs. control,

# p < 0.05 vs. low volume,

§ p < 0.05 vs. respective group without L-NAME and

† p < 0.05 vs. control + L-NAME.

### Acute effect of different resistance exercise volumes on the endothelial NO synthesis

**Figure 2:**
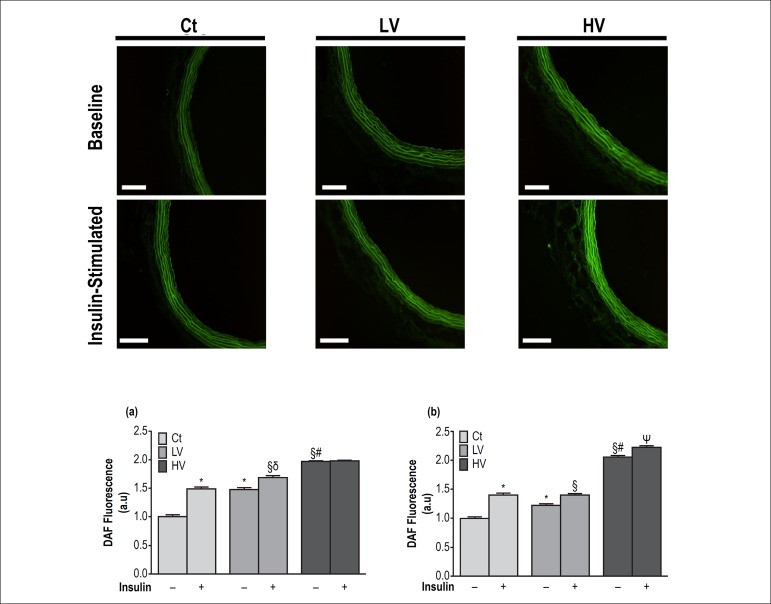
Effects of a single bout of resistance exercise in different levels of volume on endothelium-derived nitric oxide production. Detection of nitric oxide by DAF (diaminofluorescein) fluorescence at baseline and after stimulation with insulin (10 nM) (top). (a) Quantitative analyses of DAF fluorescence in endothelial layer before and after insulin stimulation; (b) quantitative analyses of DAF fluorescence in smooth muscle layer before and after insulin stimulation. Scale: 20*µ*m. Ct: control, LV: low-volume and HV: high-volume. Statistical differences were determined by one-way ANOVA followed by Bonferroni's test. Results are expressed as mean ± SEM. *p < 0.05 vs. Ct(-); ^§^p < 0.05 vs. LV(-); ^δ^p < 0.05 vs. Ct (+); ^#^p < 0.05 vs. LV(+); Ψp < 0.05 vs. HV(-).

Interestingly, under basal condition, there was a significant volume-dependent increase in NO production in endothelium and smooth muscle layer (Figure 2a and 2b). After insulin stimulation, we found an enhanced endothelial NO production in Ct and LV, but not in HV when compared to their respective group under basal conditions (Figure 2a). On the other hand, in smooth muscle layer, NO fluorescence was significantly increased in all groups when compared to their respective group at baseline (Figure 2b). However, it is important to highlight that insulin-stimulation in the LV group reached similar DAF fluorescence level as the Ct group (Figure 2b).

**Figure 3:**
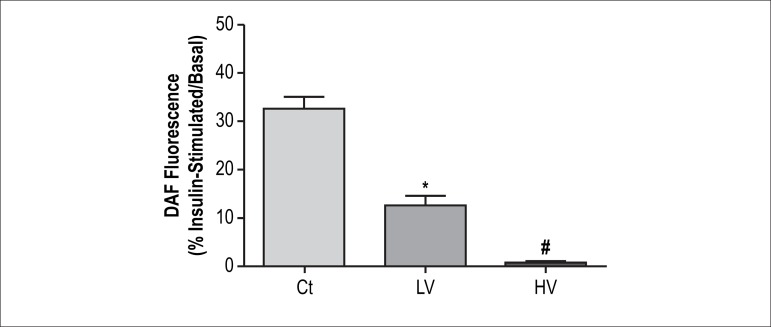
Effect of a single bout of resistance exercise in different levels of volume on additional NO synthesis in mesenteric artery stimulated by insulin (10 nmol/L). The graph shows the ratio of NO fluorescence in insulin-stimulated rings and NO fluorescence at baseline. Ct: control, LV: low-volume and HV: high-volume. Statistical differences were determined by one-way ANOVA followed by Bonferroni's test. Results are expressed as mean ± SEM. *p < 0.05 vs. Ct and ^#^p < 0.05 vs. LV.

In order to precisely evaluate the global increase in NO synthesis, the endothelial and smooth muscle layer fluorescence data were pooled and normalized to their respective group under basal conditions. The results were expressed as percentage of increase in comparison of baseline. In this experimental approach, we found a reduction in the additional production of NO in a volume-dependent way (Figure 3).

## Discussion

In the present study, we demonstrated that one single bout of different volumes but same intensity of resistance exercise promotes acute endothelial adaptations in healthy animals in a volume-dependent way. The animals subjected to 15 sets / 10 repetitions (HV group) had a more pronounced vasodilatory response. In summary, our results indicate that high volume of resistance exercise promotes an improvement in the arterial relaxation induced by insulin due an enhanced NO production.

Insulin is well known to exert a crucial role in the maintenance of metabolic homeostasis; however, this hormone also plays a key role in the cardiovascular system. In vascular bed-specific endothelial cells, insulin causes a rapid and concentration-dependent increase in the production of NO through the activation of endothelial NO synthase.^16,17^ In our study, low-volume of resistance exercise was not able to promote an increase in the vascular relaxation. On the other hand, high-volume of resistance exercise produced an increased insulin-induced vasodilation in superior mesenteric artery. Similarly, Mota et al.^13^ observed that high-intensity resistance exercise enhanced the relaxation induced by insulin in mesenteric artery of healthy animals. Thus, we hypothesize that both, high-volume and -intensity, are linked with enhanced vascular function.

To understand the participation of NO synthase in the insulin-induced relaxation, we performed concentration-response curves for insulin in pre-incubated vascular rings with L-NAME. Our data showed that insulin-induced relaxation was fully abolished by L-NAME in all groups, reinforcing the great contribution of NO in the arterial relaxation promoted by insulin.

In addition, our group has previously reported insulin-induced vasoconstriction in exercised animals via activation of MAPK/endothelin-1 pathway. This corroborates our finding on the contraction response in NO synthase inhibition in animals submitted to a single section of resistance exercise. Thus, as previously reported by our group, the functional interaction between these intracellular signaling pathways plays an essential role in the regulation of the myogenic tone in the vasculature.^12^

Our *in situ* results of NO production in superior mesenteric artery of exercised animals at different volumes demonstrated a volume-dependent increase of NO production in the endothelium and smooth muscle layers. Interestingly, in mesenteric artery rings stimulated with insulin, the additional synthesis of NO was lower in exercised animals than in the Ct group. Furthermore, our data showed that the exercised groups already had increased baseline levels of NO, and hence it is reasonable to suggest that resistance exercise might increase NO synthase activity to a sub-maximal level, preventing a substantial increase of NO synthesis in insulin-stimulated mesenteric rings.

Indeed, to exert its biological effects, endothelium-derived NO must reach the underlying smooth muscle cells.^5^ Although the time-dependent diffusion rate of NO across the cell membrane is poorly understood, new molecular players have been described to be involved on the NO transport mechanisms.^18^ Studies have consistently suggested that NO activates and permeates hemichannels formed by connexins (Cxs 37, 40 or 43) which are required to transfer NO from endothelial to smooth muscle cells. Therefore, differently from the HV group, in which a significant increase in vasodilation was observed, the existence of a positive trend but not significant in the LV group may be explained, at least in part, by the achievement of only suboptimal levels of NO in the smooth muscle cells. In addition, despite this mechanism was not investigated in the present study, further studies should evaluate whether resistance exercise improves gap junction channel function, and subsequently, promotes vasodilation. 

Several studies using a single bout of aerobic^19^ or resistance^20,21^ exercise observed an enhanced vascular relaxation, suggesting an increased NO bioavailability after a session of exercise. Furthermore, the role of resistance exercise has been evaluated in the prevention and treatment of several cardiovascular diseases.^20,22^ However, although the majority of the studies focus on the vascular effects of aerobic exercise, our data are the first that demonstrate the volume-dependent effect on vascular adaptations after a single bout of resistance exercise. Finally, the current findings may contribute to the establishment of safe limits of exercise for patients with endothelial dysfunction and insulin resistance.

## Conclusion

In summary, we demonstrated that a single bout of resistance exercise is able to improve insulin-induced vasodilation and increase NO production in a volume-dependent manner in healthy animals. Therefore, our results suggest that vascular response to resistance exercise is directly related its volume and, hence, high-volume exercise plans should be further investigated in the treatment of cardiovascular diseases and/or maintenance of a healthy life.

## References

[1] Newcomer SC, Thijssen DH, Green DJ (2011). Effects of exercise on endothelium and endothelium/smooth muscle
cross talk: role of exercise-induced hemodynamics. J Appl Physiol (1985).

[2] Malfatto G, Facchini M, Sala L, Branzi G, Bragato R, Leonetti G (1998). Effects of cardiac rehabilitation and beta-blocker therapy on
heart rate variability after first acute myocardial
infarction. Am J Cardiol.

[3] El-Sayed MS, Sale C, Jones PG, Chester M (2000). Blood hemostasis in exercise and training. Med Sci Sports Exerc.

[4] Fernandes AA, Faria TO, Ribeiro Junior RF, Costa GP, Marchezini B, Silveira EA (2015). A single resistance exercise session improves myocardial
contractility in spontaneously hypertensive rats. Braz J Med Biol Res.

[5] Ignarro LJ (1989). Endothelium-derived nitric oxide: actions and
properties. FASEB J.

[6] Walløe L, Wesche J (1988). Time course and magnitude of blood flow changes in the human
quadriceps muscles during and following rhythmic exercise. J Physiol.

[7] Joyner MJ, Casey DP (2015). Regulation of increased blood flow (Hyperemia) to muscles during
exercise: a hierarchy of competing physiological needs. Physiol Rev.

[8] Laughlin MH (1987). Skeletal muscle blood flow capacity: role of muscle pump in
exercise hyperemia. Am J Physiol.

[9] Blanco-Rivero J, Roque FR, Sastre E, Caracuel L, Couto GK, Avendaño MS (2013). Aerobic exercise training increases neuronal nitric oxide release
and bioavailability and decreases noradrenaline release in mesenteric artery
from spontaneously hypertensive rats. J Hypertens.

[10] Balon TW, Nadler JL (1994). Nitric oxide release is present from incubated skeletal muscle
preparations. J Appl Physiol (1985).

[11] Roberts CK, Barnard RJ, Jasman A, Balon TW (1999). Acute exercise increases nitric oxide synthase activity in
skeletal muscle. Am J Physiol.

[12] Fontes MT, Silva TL, Mota MM, Barreto AS, Rossoni LV, Santos MR (2014). Resistance exercise acutely enhances mesenteric artery
insulin-induced relaxation in healthy rats. Life Sci.

[13] Mota MM, Mesquita TR, Silva TL, Fontes MT, Lauton-Santos S, Capettini LS (2015). Endothelium adjustments to acute resistance exercise are
intensity-dependent in healthy animals. Life Sci.

[14] Tamaki T, Uchiyama S, Nakano S (1992). A weight-lifting exercise model for inducing hypertrophy in the
hindlimb muscles of rats. Med Sci Sports Exerc.

[15] Macedo FN, Mesquita TR, Melo VU, Mota MM, Silva TL, Santana MN (2016). Increased nitric oxide bioavailability and decreased sympathetic
modulation are involved in vascular adjustments induced by low-intensity
resistance training. Front Physiol.

[16] Salt IP (2013). Examining the role of insulin in the regulation of cardiovascular
health. Future Cardiol.

[17] Muniyappa R, Montagnani M, Koh KK, Quon MJ (2007). Cardiovascular actions of insulin. Endocr Rev.

[18] Figueroa XF, Lillo MA, Gaete PS, Riquelme MA, Sáez JC (2013). Diffusion of nitric oxide across cell membranes of the vascular
wall requires specific connexin-based channels. Neuropharmacology.

[19] Sun MW, Zhong MF, Gu J, Qian FL, Gu JZ, Chen H (2008). Effects of different levels of exercise volume on
endothelium-dependent vasodilation: roles of nitric oxide synthase and heme
oxygenase. Hypertens Res.

[20] Silva TL, Mota MM, Fontes MT, Araújo JE, Oliveira Carvalho V, Bonjardim LR (2015). Effects of one resistance exercise session on vascular smooth
muscle of hypertensive rats. Arq Bras Cardiol.

[21] Faria Tde O, Targueta GP, Angeli JK, Almeida EA, Stefanon I, Vassallo DV (2010). Acute resistance exercise reduces blood pressure and vascular
reactivity, and increases endothelium-dependent relaxation in spontaneously
hypertensive rats. Eur J Appl Physiol.

[22] Araújo AJ, Santos AC, Souza KS, Aires MB, Santana-Filho VJ, Fioretto ET (2013). Resistance Training controls arterial blood pressure from L-NAME
induced hypertensive rats. Arq Bras Cardiol.

